# Editorial: Inner Experiences: Theory, Measurement, Frequency, Content, and Functions

**DOI:** 10.3389/fpsyg.2015.01758

**Published:** 2015-11-23

**Authors:** Alain Morin, Jason D. Runyan, Thomas M. Brinthaupt

**Affiliations:** ^1^Department of Psychology, Mount Royal UniversityCalgary, AB, Canada; ^2^Department of Psychology, Indiana Wesleyan UniversityMarion, IN, USA; ^3^Department of Psychology, Middle Tennessee State UniversityMurfreesboro, TN, USA

**Keywords:** resting state, mind wandering, inner speech, unsymbolized thinking, fMRI methods, thought sampling, self-report instruments

It is safe to posit that human beings have been interested in their own inner mental experiences from the moment they became aware of them, arguably over 60,000 years ago (Leary, 2004). In sharp contrast, growth in the actual scientific examination of these inner experiences is remarkably recent (e.g., Csikszentmihalyi and Figurski, 1982; Klinger and Cox, 1987–1988; Goldstein and Kenen, 1988; Hurlburt, 1990). Inner speech, in particular, has been the focus of even more recent efforts (e.g., Morin et al., 2011; Brinthaupt and Dove, 2012; Hurlburt et al., 2013; Alderson-Day and Fernyhough, 2015; Alderson-Day et al., 2015). We present 14 articles that cover theoretical ideas as well as current research results pertaining to the measurement, frequency, content, and functions of inner experiences. In what follows we summarize some exciting key findings highlighted in this research topic.

## Content and functions of inner experiences/inner speech

There are large individual differences in inner experiences (i.e., inner speech, inner seeing, unsymbolized thinking, feelings, sensory awareness). In particular, resting states (relaxing without falling asleep with eyes open) seem to differ substantially from one participant to the next (Hurlburt et al., 2015). The resting state includes several distinct dimensions such as thinking about others' mental states, planning, sleepiness, bodily awareness, inner speech, mental imagery, and health concerns (Diaz et al., 2014). Inner speech probably represents a speaking activity that does not have a proper function in cognition. Rather, it inherits the array of functions of outer speech (Martínez-Manrique and Vicente, 2015). Furthermore, the relation between inner and outer speech is more complex than initially thought—e.g., patients with inner speech deficits can still overtly name objects (Langland-Hassan et al., 2015).

## Measurement of inner experiences

Many existing self-consciousness scales measure various related—yet different—self-reflective constructs such as adaptive-maladaptive, public-private self-consciousness, and mindfulness (DaSilveira et al., 2015). Smartphone technology allows us to significantly refine current thought sampling methods (e.g., ecological momentary assessment)—e.g., by gathering repeated sampling within various situations of daily life in very large samples, and allowing the capture of dispositional expressions (Runyan and Steinke, 2015). Repeated sampling can also promote self-awareness and mindfulness. Also, inner speech measured with self-report questionnaires and thought sampling procedures poorly correlate, suggesting that self-report approaches may not be valid (Alderson-Day and Fernyhough, 2015; also see Uttl et al., 2011). One exception is the Self-Talk Scale, which exhibits good psychometric qualities in multiple studies (Brinthaupt et al., 2015).

## Memory, time perception, aesthetic experience, and imagination

When compared to younger participants, older adults report more details (e.g., when and where of events, emotions experienced, people and objects involved) in their remote/recent autobiographical memories (Gardner et al., 2015). Delayed video presentations of one's own body image alters time perception (Fritz et al., 2015). Aesthetic insight (i.e., the “aha” phenomenon) occurs as artistic material becomes more complex and determinate, increases liking, and is preceded by increased interest, supporting the theory that interest is increased by the expectation of understanding (Muth et al., 2015). Imagination and creative thought likely emerge from the conceptual and factual knowledge accumulated throughout our lives—our semantic learning (Abraham and Bubic, 2015).

Based on the 14 articles presented here, we suggest that future work on inner experiences expand on (a) future-oriented thinking, (b) naturally occurring mentalizing, (c) mindfulness, (d) abnormal manifestations of inner experiences, (e) correlations between inner experiences and actual behavior, (f) inner experience changes with age, (g) inner experiences in altered states of mind and religious states, as well as (h) in infants and non-human animals.

## Author contributions

AM wrote the editorial; JR and TB edited it.

### Conflict of interest statement

The authors declare that the research was conducted in the absence of any commercial or financial relationships that could be construed as a potential conflict of interest.
